# Detection of Diabetic Macular Edema in Optical Coherence Tomography Image Using an Improved Level Set Algorithm

**DOI:** 10.1155/2020/6974215

**Published:** 2020-04-30

**Authors:** Zhenhua Wang, Wenping Zhang, Yanan Sun, Mudi Yao, Biao Yan

**Affiliations:** ^1^College of Information Science, Shanghai Ocean University, Shanghai 201306, China; ^2^Eye Institute, Eye and ENT Hospital, Shanghai Medical College, Fudan University, Shanghai 200030, China; ^3^The Affiliated Eye Hospital, Nanjing Medical University, Nanjing, China

## Abstract

Diabetic macular edema (DME) is a major cause of visual loss in the patients with diabetic retinopathy. DME detection in Optical Coherence Tomography (OCT) image contributes to the early diagnosis of diabetic retinopathy and blindness prevention. Currently, DME detection in the OCT image mainly relies on the handwork by the experienced clinician. It is a laborious, time-consuming, and challenging work to organize a comprehensive DME screening for diabetic patients. In this study, we proposed a novel algorithm for the detection and segmentation of DME region in OCT image based on the K-means clustering algorithm and improved Selective Binary and Gaussian Filtering regularized level set (SBGFRLS) algorithm named as SBGFRLS-OCT algorithm. SBGFRLS-OCT algorithm was compared with the current level set algorithms, including C-V (Chan-Vese), GAC (geodesic active contour), and SBGFRLS, to estimate the performance of DME detection. SBGFRLS-OCT algorithm was also compared with the clinician to estimate the precision, sensitivity, and specificity of DME segmentation. Compared with C-V, GAC, and SBGFRLS algorithm, the SBGFRLS-OCT algorithm enhanced the accuracy and reduces the processing time of DME detection. Compared with manual DME segmentation, the SBGFRLS-OCT algorithm achieved a comparable precision (97.7%), sensitivity (91.8%), and specificity (99.2%). Collectively, this study presents a novel algorithm for DME detection in the OCT image, which can be used for mass diabetic retinopathy screening.

## 1. Introduction

Diabetic retinopathy (DR) is one of the most common complications of diabetes mellitus [1]. The prevalence of DR in diabetic patients is expected to be over 20% globally [2]. Diabetic patients can develop diabetic macular edema (DME) with the progression of DR [3]. DME is caused by the accumulation of fluid in the macula due to the disrupted blood-retinal barrier [4]. It is usually recognized as the primary cause of vision loss in DR [5]. DME can be cured if they were detected at the early stage. However, due to the ignorance and unawareness especially in rural areas, many people are suffering from DME, which eventually leads to irreversible blindness [6].

Optical coherence tomography (OCT) is a noninvasive imaging modality, which provides the morphological tissue information, including the retina [2, 7]. Retinal OCT image provides information about retinal internal structures and early symptoms of retinal disease [8]. Moreover, OCT can be used for detecting the DME region [9–12]. However, it is a time-consuming and subjective task to detect DME region due to the inhomogeneous appearance, considerable shape variability, as well as the intensity similarity between DME region and healthy region [13]. Moreover, the patients with diabetes require regular and repetitive retinal screening for early detection and timely treatment of DR [14, 15]. Notably, the number of people with diabetes has risen sharply in recent years [16]. The task for comprehensive screening to detect DME in diabetic patients is very challenging [6, 17]. Thus, it is required to design a simple method to detect DME in the OCT image, which can assist ophthalmologists for DME recognition and enhance the efficiency of diagnosis and decision making [18, 19].

Compared with the rapid development of OCT technology, the method for OCT image analysis has begun during the last decade. Several algorithms, such as Geodesic active contour (GAC) and Chan-Vese (C-V), have been used for medical image analysis. GAC algorithm usually leads to a high level of noise. The object is usually characterized by weak edges [20]. C-V model is time-consuming because the average intensities inside and outside the contour should be computed at each iteration [21]. These algorithms are only appropriate for the limited number of images with the determinate abnormalities [22]. In this study, we proposed a novel algorithm for the delineation of the DME region in the OCT image. The algorithm is based on the K-means clustering algorithm and the improved SBGFRLS level set algorithm. This algorithm will assist ophthalmologists for DME segmentation and enhance the efficiency of DR diagnosis.

## 2. Material and Methods

### 2.1. Dataset

This study was conducted in compliance with the tenets of the Declaration of Helsinki and approved by the ethics committee of the author's institute. Inclusion criteria were as follows: presence of macular edema in at least one eye and clear optical media allowing OCT imaging in high quality. If a patient's compliance permitted, both eyes were eligible for inclusion.

The OCT dataset contains 100 OCT images (10 normal images and 90 DR images). The normal OCT images were from 10 healthy volunteers. The OCT images with DME regions were from 80 DR patients (30 females and 50 males) who undergone spectral domain OCT scanning (Heidelberg Engineering, Heidelberg, Germany). Each patient was scanned at the same devices by the same operator to avoid the potential systematic bias. All SD-OCT images were read and assessed by trained graders and identified as normal or DME based on the evaluation of retinal thickening, hard exudates, intraretinal cystoid space formation, and subretinal fluid. The following OCT images were ruled out for further analysis: (1) low contrast of OCT images makes the interface between the background region and retinal region is quite weak and invisible; (2) OCT images with high speckle noise hinders DME signal in retinal region.

### 2.2. Methodology of Diabetic Macular Edema Detection

**Figure 1:**
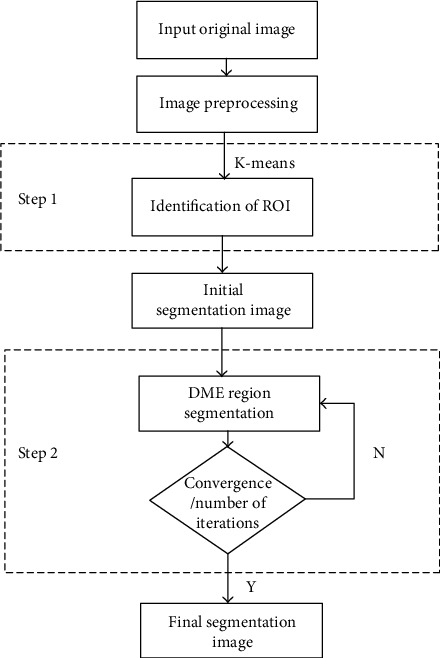
Flow chart of the proposed methodology for DME segmentation.

The methodology consists of two steps. At step 1, the K-means clustering algorithm is used to divide the input OCT image into the ROI (retina region) and background region. At step 2, the improved SBGFRLS algorithm is used to segment the DME region in the OCT image. The flowchart of the methodology is shown in Figure 1.

**Step 1 .**

Original OCT images are divided into the ROI (retinal region) and background region.

K-means clustering algorithm is a common image segmentation algorithm based on the clustering technique [23]. The core of the algorithm is to determine K clustering centers *C*_1_, *C*_2_, ⋯, *C*_*K*__,_ so as to minimize the mean squared distance from each data pint to its nearest center. In this study, K = 2 was used to divide OCT image into the ROI region and background region.

**Step 2 .**

Improved SBGFRLS algorithm is used for DME segmentation in OCT image.

The level set methods are usually used for medical image segmentation, including the Selective Binary and Gaussian Filtering Regularized Level Set (SBGFRLS) [24], Chan-Vese (C-V) [25], and Geodesic Active Contour (GAC) algorithm [26]. In this study, we modified the SBGFRLS algorithm to propose a novel algorithm for DME segmentation in OCT image, named as SBGFRLS-OCT. SBGFRLS algorithm is used to segment OCT image by calculating the signed pressure force function (spf) and reinitialization [27]. The evolution function of SBGFRLS algorithm is

(1) $$\begin{array}{c} \frac{\partial \phi }{\partial t}=\text{spf}\left(I\left(x\right)\right)\left|\nabla \phi \right|\alpha , \end{array}$$

where *α* is the force parameter of the balloon and controls the evolution rate of the level set. The function of spfis defined as:

(2) $$\begin{array}{c} \text{spf}\left(I\right)=\frac{I-\left(\left(c_{1}+C\right)/2\right)}{\max\left(\left|I-\left(\left(c_{1}+C\right)/2\right)\right|\right)}, \end{array}$$

where *c*_1_ and *c*_2_represent the mean gray value of inside and outside the curve, respectively, which is computed by Eq. (3).

(3) $$\begin{array}{c} \left\{\begin{array}{c} c_{1}\left(\phi \right)=\frac{\int_{\Omega }I\left(x,y\right)\cdot H\left(\phi \right)dxdy}{\int_{\Omega }H\left(\phi \right)dxdy},\\ c_{2}\left(\phi \right)=\frac{\int_{\Omega }I\left(x,y\right)\cdot \left(1-H\left(\phi \right)\right)dxdy}{\int_{\Omega }\left(1-H\left(\phi \right)\right)dxdy}. \end{array}\right. \end{array}$$

We modified the spf function of the conventional SBGFRLS algorithm in the proposed SBGFRLS-OCT algorithm. The SBGFRLS-OCT algorithm is efficient to obtain the segmentation result of the internal closed region such as the DME region, whereas the conventional SBGFRLS algorithm cannot achieve. Here, we provided a detailed description of SBGFRLS-OCT algorithm as shown below:

1. Calculate the outside curve of the retinal region (C) by K-means clustering algorithm
2. Taking C as an input parameter, calculated the inside curve (c_1_) by the modified spf function, as shown in Eq. (4)

(4) $$\begin{array}{c} \text{spf}\left(I\right)=\frac{I-\left(\left(c_{1}+C\right)/2\right)}{\max\left(\left|I-\left(\left(c_{1}+C\right)/2\right)\right|\right)}. \end{array}$$

(3) If the level set function converges, it ends; otherwise, it returns (2).

## 3. Results

### 3.1. Performance of SBGFRLS-OCT Algorithm on ROI and DME Segmentation

**Figure 2:**
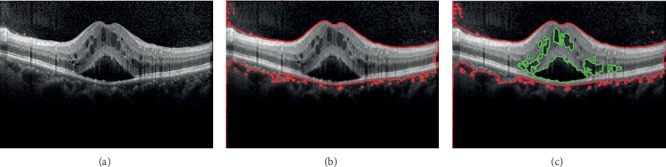
Original OCT image was segmented to obtain the ROI region and DME region. (a) Original OCT image; (b) K-means clustering is used for ROI segmentation; (c) SBGFRLS-OCT algorithm is used for DME segmentation. Red lines were used to label the retinal regions (ROI), and green lines were used to label DME regions.

We used the SBGFRLS-OCT algorithm for DME segmentation in the OCT image.  Figure 2 shows a representative segmentation result of the OCT image. At first, the original input OCT image (Figure 2(a)) was segmented to obtain the ROI region and background region based on the K-means clustering algorithm (Figure 2(b)). Then, the ROI region was segmented to a complete segmentation result of ROI and DME region based on the SBGFRLS-OCT algorithm (Figure 2(c)).

**Figure 3:**
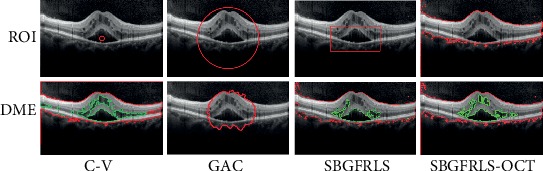
Comparison of segmentation performance of the SBGFRLS-OCT algorithm against C-V, GAC, and SBGFRLS algorithms. DME segmentation in the OCT image was conducted using C-V, GAC, SBGFRLS, and SBGFRLS-OCT algorithms to obtain ROI and DME region. The four images showed the segmentation results. Red lines were used to mark the retinal region (ROI). Green lines were used to mark the DME regions.

We then compared the segmentation performance of the SBGFRLS-OCT algorithm against three conventional level set algorithms, including C-V, GAC, and SBGFRLS. As shown in Figure 3 the C-V algorithm could not obtain the initial boundary of ROI. GAC algorithm could not obtain the clear segmentation result of the ROI and DME region. The traditional SBGFRLS algorithm was highly dependent on the initial boundary of ROI, which was determined by the outside curve. However, the outside curve was variable. By contrast, the SBGFRLS-OCT algorithm could obtain a perfect segmentation result as shown by the clear ROI region and DME region. Collectively, these results suggest that the SBGFRLS-OCT algorithm is suitable for DME segmentation in the OCT image.

### 3.2. Comparison of DME Segmentation Efficiency between SBGFRLS-OCT Algorithm and Other Level Set Algorithms

We calculated the processing time and the number of iteration times to compare DME segmentation efficiency between SBGFRLS-OCT and other level set algorithms. As shown in Table 1, compared with the SBGFRLS algorithm, the processing time of the SBGFRLS-OCT algorithm reduced by 30%, while the number of iteration time reduced by 35%. Compared with C-V or GAC algorithm, the SBGFRLS-OCT algorithm greatly reduced the processing time for DME segmentation. The processing time in the SBGFRLS-OCT algorithm was about 90 times or 275 times less than the time in C-V or GAC algorithm. The SBGFRLS-OCT algorithm greatly reduced the number of iteration times. The number of iteration times in the SBGFRLS-OCT algorithm was about 73 times or 300 times less than the number in C-V or GAC algorithm.

### 3.3. Comparison of DME Segmentation Performance between SBGFRLS-OCT Algorithm and Manual Method

**Figure 4:**
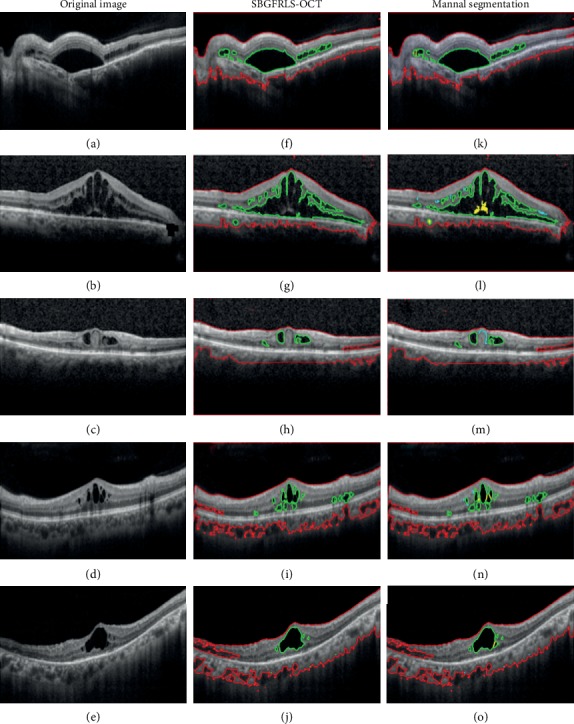
Comparison of DME segmentation performance between the SBGFRLS-OCT algorithm and manual method. (a–e) Original OCT images with DME pathology. (f–j) DME segmentation result by SBGFRLS-OCT algorithm. (k–o) Manual segmentation result by five different clinicians. Red lines were used to label the retinal region (ROI). Green lines were used to label DME regions.

90 OCT images with DME region were carefully judged by three retinal experts with more than 10-year clinical experience. Their consistent results of DME segmentation were taken as the gold standard. We then compared the performance of DME segmentation between the SBGFRLS-OCT algorithm and manual method by 5 additional clinicians by calculating the indicators, including Dice's similarity coefficient, precision, sensitivity, and specificity [16]. It is generally accepted that the Dice's similarity coefficient greater than 0.70 indicates excellent agreement. The Dice's similarity coefficient could reach 0.97 in the SBGFRLS-OCT algorithm, suggesting that the SBGFRLS-OCT algorithm can achieve similar segmentation performance as a manual method.  Table 2 showed that the precision, sensitivity, and specificity for DME segmentation were 97.7%, 91.8%, and 99.2% in the SBGFRLS-OCT algorithm, suggesting that the SBGFRLS-OCT algorithm has a comparable performance as manual segmentation for DME segmentation. By contrast, the processing time of the SBGFRLS-OCT algorithm was about 66 times less than the time of manual segmentation.  Figure 4 shows the segmentation results of the DME region between the SBGFRLS-OCT algorithm and manual segmentation.

## 4. Discussion

Diabetic macular edema (DME) is a major cause of blindness in the patients with diabetic retinopathy [28]. Optical coherence tomography (OCT) has gained increasing attention as a diagnosis tool for DME detection [29]. However, comprehensive OCT screening for DME in diabetic patients is a labor-intensive, tedious, and challenging task [30]. Thus, it is urgent to develop a rapid and simple algorithm for DME segmentation in OCT images.

The level set algorithms have been used for medical image segmentation [31]. However, clinical specialists are often overwhelmed by the intensive computational requirements and complex regulation of controlling parameters [32]. K-means clustering algorithm is simple and has a relatively low computational complexity. It can obtain the approximate boundaries of potential components of interest [33, 34]. The improved SBGFRLS algorithm enhances the efficiency and precision for DME segmentation. Compared with the current level set algorithms, such as C-V, GAC, and SBGFRLS, the proposed SBGFRLS-OCT algorithm is more efficient in DME segmentation as shown by decreased mean processing time (s) and iteration times (time). Dice's similarity coefficient is a dimensionless ratio where 1 corresponds to a perfect match between the images being compared. Dice's similarity coefficient > 0.70 indicates excellent agreement [16]. The Dice's similarity coefficient can reach 0.97 in the SBGFRLS-OCT algorithm. The precision, sensitivity, and specificity of DME segmentation in the SBGFRLS-OCT algorithm can reach about 97.7%, 91.8%, and 99.2%. Compared with manual segmentation, the SBGFRLS-OCT algorithm can significantly reduce the processing time of DME segmentation, suggesting that the SBGFRLS-OCT algorithm is comparable to retinal specialists in DME segmentation.

Given the wide variations in pathology and the potential fatigue of human experts, researchers and doctors have begun to use computer-assisted interpretation of OCT image [2, 4]. Deep learning has become a methodology of choice for analyzing OCT images. It helps to identify, classify, and quantify pathological features in OCT images. However, deep learning is highly dependent on the following factors: (a) advances in high-tech central processing units (CPUs) and graphics processing units (GPUs), (b) the availability of a huge amount of data (i.e., big data), and (c) developments in learning algorithms [35, 36]. However, in the hospitals of rural areas, they do not have high configuration computers and sophisticated computer specialists. A simple and easily implemented method is required for OCT image analysis. Compared with deep learning, the SBGFRLS-OCT algorithm is simple and does not require multiple training images and complexed computational devices. Moreover, the SBGFRLS-OCT algorithm obtains similar results of OCT image analysis as the physicians.

In the clinical work, the quality of OCT images was influenced by multiple factors, such as OCT devices, OCT operators, and disease conditions [37]. For example, cataract-induced lens opacity significantly affected the quality of the OCT image [38]. The interpretation result of the OCT image may also be influenced by the quality of OCT images. The SBGFRLS-OCT algorithm would face great challenges when it was used for DME segmentation of the following type of OCT images: (1) low contrast of OCT images makes the interface between the background region and retinal region is quite weak and invisible; (2) OCT images with high speckle noise hinders DME signal in retinal region. In the future, we would modify the SBGFRLS-OCT algorithm by integrating the de-noise algorithm and image intensification algorithm and make it applicable to the interpretation of OCT images with different quality.

## 5. Conclusions

Overall, in this study, a novel algorithm was proposed for DME segmentation based on the K-means clustering algorithm and improved SBGFRLS algorithm. Compared with the other level set algorithms, the SBGFRLS-OCT algorithm has higher precision, higher sensitivity, and better specificity for DME segmentation. Compared with manual DME segmentation, the SBGFRLS-OCT algorithm achieves a comparable precision, sensitivity, and specificity but a significantly reduced processing time. Collectively, this study presents a novel algorithm for DME segmentation in the OCT image, which can be used for mass diabetic retinopathy screening.

## Figures and Tables

**Table 1:** Mean processing time and iteration times for DME segmentation by C-V, GAC, SBGFRLS, and SBGFRLS-OCT.

	C-V	GAC	SBGFRLS	SBGFRLS-OCT
Processing time (s)	2068.25 ± 198.59^∗^	6362.72 ± 809.77^∗^	33.24 ± 4.13^∗^	23.32 ± 3.86
Iteration times (time)	13248 ± 989.77^∗^	53563 ± 1482.53^∗^	280.58 ± 23.38^∗^	180.32 ± 14.28

All data were shown as mean ± SD. *n* = 90. The significant difference was calculated by one-way ANOVA. ^∗^*P* < 0.05 versus SBGFRLS-OCT.

**Table 2:** Comparison of segmentation performance between SBGFRLS-OCT algorithm and five different clinicians.

	Precision	Sensitivity	Specificity	Processing time (s)
Clinician 1	95%	88%	96%	1580
Clinician 2	92%	85%	93%	1200
Clinician 3	98%	94%	99%	2000
Clinician 4	94%	90%	93%	1680
Clinician 5	90%	95%	91%	1880
Average value for clinician	94% ± 3%	90% ± 4%	94% ± 3%	1668 ± 309
SBGFRLS-OCT	97.7%	91.8%	99.2%	25

## Data Availability

The data used to support the findings of this study are included within the article.
